# Quantification of the Effect of the Cattle Breed on Milk Cheese Yield: Comparison between Italian Brown Swiss and Italian Friesian

**DOI:** 10.3390/ani10081331

**Published:** 2020-08-01

**Authors:** Piero Franceschi, Massimo Malacarne, Paolo Formaggioni, Michele Faccia, Andrea Summer

**Affiliations:** 1 Department of Veterinary Science–University of Parma, Via del Taglio 10, I-43126 Parma, Italy; piero.franceschi@unipr.it (P.F.); paolo.formaggioni@unipr.it (P.F.); andrea.summer@unipr.it (A.S.); 2 Department of Soil, Plant and Food Sciences, University of Bari, Via Amendola 165/A, 70125 Bari, Italy; michele.faccia@uniba.it

**Keywords:** Parmigiano Reggiano cheese, cheese yield prediction formula, multiple regression analysis, k-casein B

## Abstract

**Simple Summary:**

It is well known that cattle breeds can produce milk with significant differences in casein and fat contents that are reflected in higher or lower cheese yields. However, the different cheese-yielding ability also involves some particular breed-related molecular characteristics that are not yet considered in milk quality payment systems, due to difficulties in their measurement. The aim of this research was to propose a method for the comprehensive quantification of the effect of milk characteristics on the cheese-making efficiency, including those connected to molecular peculiarities, in terms of casein units. In particular, the method was applied to Parmigiano Reggiano cheese by comparing two different cattle breeds, Italian Friesian and Italian Brown. Under the same processing conditions, the cheese-making efficiency of Italian Brown was higher than that of Italian Friesian. The study concluded that the added value of Italian Brown milk can be expressed in terms of +0.20 g/100 g casein. The method developed could be easily used at the dairy farm level.

**Abstract:**

Milk from different cattle breeds can present different casein and fat contents, which are reflected in different cheese yields (CY). However, CY is also related to some breed-related molecular characteristics. The aim of the present work was to quantify the effect of these characteristics by comparing a series of Parmigiano Reggiano (PR) cheese-making trials made with milks from Italian Brown (IB) and Italian Friesian (IF) cattle herds. Twelve trials were carried out in a cheese factory in one year (one trial per month), each one consisting of four vats processed in parallel: three vats contained milk from three different IF cattle herds (IF1, IF2 and IF3) and one contained milk from a single IB cattle herd. A 24-h CY prediction formula was developed with data from IF1, IF2 and IF3 trials (calibration) and successively validated by applying it to 12 PR trials made with IF milk in six different cheese factories (external validation). The predicted values of 24-h CY were no different to the actual ones in both calibration and external validation. Finally, the formula was tested on trials made with IB milk. In this case, the predicted values were lower than the actual ones. The quantity of IF milk casein necessary to give the same CY of IB milk was 0.20 g/100 g.

## 1. Introduction

The profitability of the dairy industry is closely linked to the cheese yield capacity of the milk processed, expressed as the kg of cheese obtained from 100 kg milk. As the cheese-making process basically consists of the concentration and following partial dehydration of the casein matrix in which fat globules are entrapped (the curd), the cheese yield is positively correlated with the casein and fat contents of milk. Of course, the yield is also influenced by the processing conditions that determine the amount of whey retained in cheese, such as the rennet dose, pH of vat milk, temperature of coagulation, curd cutting and cooking, pressing, etc. For this reason, fat and casein contents are greatly considered by dairies in the payment systems of milk according to quality [1]. Recently, Formaggioni et al. [2] proposed a multiple regression analysis for the development of a yield prediction formula for Parmigiano Reggiano cheese at 24 h after the extraction from the vat, using the values of casein and the fat of vat milk as predictors.

It is well known that cattle breeds can present relevant differences in milk casein and fat contents [3,4,5]. For instance, Italian Friesian cows, whose population corresponds to about 80% of dairy cows raised in Italy, produce milk with lower fat (−0.21 or −1.35 g/100 g) and casein (−0.27 or −0.38 g/100 g) contents than milks of Brown Swiss [4,5] or Jersey [3]. However, differences among breeds also include other specific properties such as the ratio among the casein fractions, genetic variants, level of post translational modifications and content of calcium phosphate [5,6,7]. All these “molecular” aspects influence the structural characteristics of the casein micelle, the aptitude of milk to rennet coagulation and the rheological properties of the resulting curd [8,9,10].

In particular, casein micelles rich in k-casein are smaller than those with a lower content of this fraction, and show optimal behavior during rennet coagulation with a better retention of fat in the para-caseinate network [6,11,12]; another relevant aspect is the presence of the B genetic variant of k-casein, which has been reported to improve the coagulation aptitude [13]. Overall, these molecular features exert an additive effect on cheese yield that goes beyond the simple “richness” in casein and fat [5]. The measurement of these properties requires time-consuming and expensive methods, such as liquid chromatography for the assessments of genetic variants of caseins, their relative proportions and the level of post-translational modifications, ultrafiltration for the measurements of salt distribution and the calcination of milk and milk fractions as a preliminary step in the measurement of the content of minerals by Atomic Absorption Spectrometry (AAS). These methods can hardly be applied as routine analyses by commercial laboratories involved in a milk quality payment system and, thus, the additive effect of molecular features is not considered.

The aim of this research is to propose a method for the comprehensive quantification of the effect of these specific characteristics of milk on the cheese-making efficiency, measured in terms of cheese yield. In particular, the study was applied to Parmigiano Reggiano cheese by comparing two different cattle breeds, Italian Friesian and Italian Brown.

## 2. Materials and Methods

### 2.1. Experimental Design

Twelve cheese-making trials were carried out at the same cheese factory for one year (1 trial per month) during the processing of milk into Parmigiano Reggiano, a cheese with a Protected Designation of Origin (PDO). In each trial, 4 vats containing milk from single herds were processed in parallel: 3 of them contained milk from Italian Friesian herds (coded IF1, IF2 and IF3) and the remaining one contained milk from a single Italian Brown herd (IB). In all herds selected, cows were kept in a loose housing system with cubicles and milked two times a day; their feeding followed the rules established by the Parmigiano Reggiano Consortium (https://www.parmigianoreggiano.com).

Cheesemaking was performed according to the official PDO protocol [14]. In short, the raw matter (about 1100 kg per vat, from now on defined as V-milk) was prepared by mixing the evening milk, partially skimmed overnight by the gravity separation of fat globules, and the full cream morning milk (about 1:1) [15]. V-milk was heated to 32 °C and about 3% of a natural whey starter was added; the starter was obtained by the spontaneous acidification of the whey from the previous day [16]. Then, calf rennet was added for coagulation (coagulation time required: 8–10 min, at pH 6.2–6.4) and the resulting coagulum was cut until the particles reached the size of a rice grain. The vat temperature was then raised to about 55 °C while the curd grains were continuously stirred (cooking phase); then, the curd grains were left to settle at the bottom of the vat, where they aggregated spontaneously to form the curd mass, for about 1 h. The curd was then extracted from the vat and divided into twin cheese wheels, which were periodically rotated over the next two days, then immersed in saturated brine for about 20 days. Finally, the cheeses entered the ripening phase for at least 12 months, even though it is commonly prolonged to 24–36 months.

For each trial, the volume of V-milk was measured by a volumetric pump and a small sample was collected before the addition of the starter. In addition, a sample of whey was collected after the extraction of the curd. Both samples, kept under refrigeration (at 4 °C), were delivered to the laboratory and immediately analyzed. Finally, the two cheese wheels were weighed 24 h after their extraction from the vat.

### 2.2. Analytical Methods

Total nitrogen (N,) non casein N and non-protein N were quantified in milk and whey by the Kjeldahl method according to the Association of Official Analytical Chemists (AOAC) standards [17,18,19] using a DK6 digestor and a UDK126A distiller unit (VELP Scientifica, Usmate, Italy). From the nitrogen data, crude protein, whey protein, casein, NPN × 6.38 and true protein were calculated. The k-casein B content in V-milk was determined by the ELISA method (Test K, Astori Tecnica, I-25020 Brescia, Italy), using a specific antibody [20], from which the k-casein B to total casein ratio was calculated. The lactose and fat content of V-milk and fat content of whey were determined by the mid-infrared method [21] using a MilkoScan FT 6000 (Foss Electric, DK-3400 Hillerød, Denmark). The dry matter of V-milk and whey was determined by oven drying at 102 °C [22], whereas the ash content was obtained after muffle calcination at 530 °C [23]. Total Ca, P and Mg in V-milk and their distribution between soluble and colloidal phases were determined according to Malacarne et al. [24] after ultrafiltration. To this purpose, an aliquot of V-milk was totally skimmed and ultrafiltered through an Amicon 8200 ultrafiltration cell equipped with a 30 KDa cutoff membrane in polyethersulfone (Merck Millipore Corporation, Darmstadt, Germany) under nitrogen flow at 75 psi. Total Ca and Mg in milk and soluble Ca, P and Mg in ultrafiltration permeate were determined in hydrochloric ash solution by Atomic Absorption Spectrometry (Perkin-Elmer 1100 B, Waltham, MA 2451, USA), whereas total P, soluble P and acid-soluble P after treatment with 12% Trichloroacetic acid were quantified by the colorimetric method of Allen [25].

Somatic cells and the total bacterial count of V-milk were determined by the fluoro-opto-electronic method [26] with the Fossomatic and flow-cytometry method using BactoScan FC (both from Foss Electric, Hillerød, Denmark), respectively. Curd fines in whey samples were measured by the gravimetric method described in a previous paper [5]. The titratable acidity and pH of V-milk were measured by titration according to the Soxhlet–Henkel method [27] and by potentiometer, respectively. The rennet coagulation parameters, clotting time (r), curd firming time (k_20_), and curd firmness (a_30_) were measured by the lactodynamographic method [28] using a Formagraph (Foss Electric, DK-3400 Hillerød, Denmark). Finally, density at 15 °C was assessed by means of a Quevenne lactometer.

### 2.3. Measures of Cheese-Making Efficiency

Actual cheese yield (ACY) was calculated according to the equation developed by Formaggioni et al. [2]:

24-h ACY = [mc (kg)] × 100/[mm (kg)],(1)

where ACY = actual cheese yield of milk (%); mc = weight of the two cheese wheels measured 24 h (24-h) after extraction from the vat; mm = weight of the V-milk processed (volume × density).

The estimated cheesemaking losses (ECL) of protein, casein and fat were calculated as follows:

ECL = (whey) × 100/(V-milk),(2)

where ECL is expressed as a percentage; (whey) is the concentration in the whey, expressed as g/100 g; (V-milk) is the concentration in the vat milk, expressed as g/100 g.

### 2.4. Cheese Yield Prediction Formula

Data collected from the trials with IF1, IF2 and IF3 milk were used as a calibration set to develop the 24-h cheese yield prediction (Formula (3), calibration phase). The method employed was a multiple regression analysis using, as predictors, the contents of fat and casein of V-milk, as described in Formaggioni et al. [2]. The following formula was obtained:

24-h PCY = 1.254 × casein + 1.426 × fat + 1.059(3)

where 24-h PCY is the predicted 24-h cheese yield value expressed as the kg of cheese obtained from 100 kg of milk; casein and fat are expressed in g/100 g V-milk.

In order to assess the reliability of the prediction formula, an external validation was carried out using data from Parmigiano Reggiano cheese-making trials with Italian Friesian milk in 6 different cheese factories (validation phase). To increase the range of variability in the validation set, two cheese-making trials were selected in each cheese factory, one with the lowest and the other with the highest casein content in V-milk. In each trial, the concentration of casein and fat in V-milk, as well as 24-h ACY, were measured as described above.

The final step was to test the formula on cheese-making trials carried out with IB milk and to quantify the effect in terms of virtual casein units of vat milk (test phase). The quantity of casein of IF milk that gave the same 24-h ACY observed in the 12 IB milk trials (CIF-casein, g/100 g) was calculated, starting from the formula of the cheese yield, as follows:

CIF-casein = (24-h ACY − 1.059 − 1.426 × fat)/1.254(4)

where 24-h ACY is the actual cheese yield of milk and fat is the concentration of this constituent in V-milk (g/100 g).

### 2.5. Statistical Analysis

The significance of the differences was tested by an analysis of variance, using the general linear model procedure of SPSS (IBM SPSS Statistics version 25, Armonk, NY 10504-1722, USA), according to the following univariate model:

Y_ijk_ = µ + H_i_ + T_j_ + ε_ijk_ (5)

where Y_ijk_ = dependent variable; µ = overall mean; H_i_ = effect of the herd (i = 1,…4); T_j_ = effect of trial (j = 1,…12); ε_ijk_ = residual error. The significance of the differences was tested by means of the Bonferroni method.

Predicted and actual 24-h cheese yield values were compared in the calibration, validation and test phases using an ANOVA univariate considering, as fixed factors, the cattle herd (3 levels in calibration: one for each IF herd) and the trial (12 levels, one for each trial, in calibration and test phases; 6 levels in validation phase). Even in this case, the significance of the differences was tested by means of the Bonferroni method.

## 3. Results and Discussion

### 3.1. Characteristic and Cheese-Yielding Ability of IB Milk and IF Milks

The chemical composition of V-milk samples is shown in Table 1. Overall, IB milk showed the highest content of dry matter, protein, whey protein, casein, k-casein B (both as grams in 100 mL milk and as % of total casein) and ash. No differences were observed among IF milks for all these parameters. The content of fat evidenced a tendency to be higher in IB milk than IF milks, as the value of *p* was >0.05 and <0.1 (*p* = 0.09). The fat to casein ratio was the same in IF milks and lower in IB milk.

Table 2 shows the content and distribution of Ca, P and Mg, as well as the content of chloride in milk. IB milk had the highest values of total, soluble and colloidal Ca, total, soluble and casein P (in the form of phosphorylated amino acids within caseins), total and colloidal Mg. In contrast, no significant differences were observed for inorganic colloidal P (in the form of calcium phosphate within the casein micelles). The values in IF milks were very similar, and only a few significant differences were observed, as for total and soluble Mg and chloride (IF3 sample had the highest level of chloride).

The values of pH, titratable acidity, rennet coagulation parameters, somatic cell and total bacterial counts are reported in Table 3. The highest pH and titratable acidity values were found in IB milk, whereas the IF samples showed similar values, except for pH in IF3, which was similar to IB milk. IF3 milk had the highest curd-firming time value and the lowest curd firmness, showing the poorest aptitude to rennet coagulation. IB milk and IF2 had a lower value of somatic cells than IF3, with IF1 showing similar values than the other V-milks.

The cheese-yielding abilities are shown in Table 4. The curd fines are curd particles lost in the whey during the vat phase of the cheese-making process (during coagulation, cutting, stirring, cooking and resting). A significant difference was only observed between IB and IF1 whey samples (lower content in IB). IB milk showed the highest ACY and the lowest ECL of fat, whereas IF milks had the same values for most parameters, except for the higher ECL of fat in IF3.

The differences observed in the chemical, protein and mineral composition between IB and IF milk are in agreement with the results reported by Malacarne et al. [29]. The k-casein B contents agree with those recently reported by Franceschi et al. [5] in a study performed on high-moisture mozzarella. Concerning the distribution of minerals, the values of colloidal Ca, P and Mg were proportional to the casein content, since they are essential constituents of the casein micelle [30].

The better aptitude for rennet coagulation of Italian Brown compared to Italian Friesian was reported by Malacarne et al. [29]; however, it depends on several factors, such as the casein content and the amount of k-casein B [13,31], while the content and the distribution of minerals are also involved. Malacarne et al. [9] reported better rennet coagulation parameters for milk with higher contents of total and colloidal Ca, P and Mg. The levels of chloride and somatic cells are positively correlated in milk, as they both increase in the case of intra-mammary infections [32]. This could explain the higher level of chloride in IF3 milk compared to other IF and IB V-milk [33].

The better cheese-making efficiency of IB milk can be attributed to several factors. The major driver was probably the high casein content, as the cheese yield is directly proportional to its concentration, which has a positive effect on the capacity of the curd to retain the other milk constituents. Another factor to be considered is the level of k-casein B, since this genetic variant is associated with improved rheological properties in the curd.

### 3.2. Quantification of the Effect of the Cattle Breed on Milk Cheese Yield

The model of 24-h PCY was significant when applied to the calibration set (*p* < 0.001) and showed values of 1.53% and 0.88 of Root Mean Square Error Prediction (RMSEP, %) and coefficient of determination (*R*^2^), respectively (Table 5). In the whole validation set, the ranges of casein concentration and 24-h ACY were 2.14–2.60 g/100 g and 7.24–8.54 kg/100 kg, respectively. Even at the two extreme points of the range, no differences between 24-h ACY and 24-h PCY were observed, despite a slight increase in RMSEP (Table 5). When the formula was tested on IB milk, the estimated values of 24-h ACY (24-h PCY) were significantly lower than the actual ones (24-h ACY) with an increase in the RMSEP value (2.94%).

Starting from the 24-h PCY formula, the quantity of IF milk casein (CIF-casein) necessary to give the same cheese yield as IB milk casein was calculated. The value obtained was higher than IB milk (3.12 vs. 2.92 g/100 g; *p* < 0.05), and the average difference (CIF-casein–IB casein) was 0.20 g/100 g casein, ranging from 0.09 to 0.49 g/100 g.

A 24-h CY prediction formula was developed with data derived from Parmigiano Reggiano (PR) cheese made with IF milk in a single cheese factory (calibration) and validated in PR cheese-making trials with IF milk in different cheese factories (external validation). This was done because Parmigiano Reggiano is an artisanal cheese made in small- to medium-sized cheese factories, where even small differences in the processing technology [16] are potentially responsible for differences in the cheese-yielding ability of the milk. The external validation confirmed the reliability of the formula to predict the 24-h cheese yield of Italian Friesian milk, even when the cheese was manufactured in different factories. On the other hand, the formula was not reliable when applied to IB milk. The higher concentrations of casein and fat in IB milk than IF milk cannot be considered as responsible for the worsening of the prediction ability of the formula, as these two variables are included in the formula. The effect of processing conditions should be considered negligible, since IB milk was processed in the same cheese factory in parallel with IF milks used to develop the formula. It is very likely that the main drivers of this effect are breed-related differences at the molecular level (for example, the highest contents of k-Cn B and minerals of IB milk reported above). The “additive effect” of these molecular characteristics on the cheese-yielding ability of milk “weighs” 0.20 g/100 g of casein, on average.

The assumption of this method of quantification is that the cheese-making efficiency of IF milk—and thus the ability of the formula to predict 24-h ACY—does not change with the casein content of V-milk when the same cheese-making technology is applied. However, the formula was calibrated in trials in which the casein values ranged within the interval 2.15–2.74 g/100 g and was tested on IB milk trials with casein contents ranging from 2.77 to 3.09 g/100 g. In actual fact, it is not possible to exclude the possibility that the cheese-making efficiency of IF milk would be similar to that of IB milk with casein in the range of 2.77–3.09 g/100 g. However, most studies report a linear and positive relationship between the values of the casein contents of vat milk and its cheese-yielding ability [2,34,35].

## 4. Conclusions

In this study, a method to quantify the effect of the breed-related molecular features on milk cheese yield in terms of casein units was developed. It could be adopted at the cheese factory level using parameters that are routinely measured such as the casein and fat content of V-milk and the weight of the cheese wheels 24 h after extraction from the vat. The “breed-related added value” may change according to the cheese factory considered, because of slight changes in the cheese-making technology.

## Figures and Tables

**Table 1:** Least square mean (LSM) values of chemical composition of vat milk from Italian Brown (IB milk) or Italian Friesian (IF1, IF2, IF3 milk) cattle herds used in 12 Parmigiano Reggiano cheese-making trials ^1^.

Parameters	Unit	IB Milk		IF1 Milk		IF2 Milk		IF3 Milk		SE ^2^	*p* ^3^
		LSM		LSM		LSM		LSM			
Dry matter	g/100 g	12.62	b	11.81	a	12.13	a	11.88	a	0.11	***
Lactose	g/100 g	4.98	b	4.85	a	4.95	b	4.84	a	0.02	***
Fat	g/100 g	3.05		2.87		2.98		2.89		0.06	NS
Ash	g/100 g	0.75	b	0.71	a	0.73	a	0.72	a	0.01	***
Crude protein	g/100 g	3.73	b	3.24	a	3.35	a	3.30	a	0.03	***
Crude whey protein	g/100 g	0.81	b	0.74	a	0.77	a	0.75	a	0.01	***
Casein	g/100 g	2.92	b	2.50	a	2.58	a	2.55	a	0.03	***
Casein number	%	78.22	b	77.13	a	77.11	a	77.26	a	0.15	***
k-casein B	g/100 mL	0.37	b	0.04	a	0.05	a	0.04	a	0.01	***
k-casein B to casein ratio	%	12.72	b	1.74	a	2.00	a	1.38	a	0.30	***
NPN × 6.38	g/100 g	0.17		0.19		0.18		0.18		0.01	NS
Fat to casein ratio	Value	1.05	a	1.15	b	1.16	b	1.13	b	0.02	***

^1^ In each trial, four cheese-making trials were performed in parallel: three with milk collected from three Italian Friesian cattle herds (IF1, IF2 and IF3) (each cheese-making was made with the milk of a single herd) and one with milk coming from a single Italian Brown cattle herd (IB). ^2^ Standard error. ^3^ *p*-value: NS. *p* > 0.05; *** *p* ≤ 0.001; ^a,b^ different for *p* ≤ 0.05.

**Table 2:** Least square mean values of mineral content and distribution of vat milk from Italian Brown (IB milk) or Italian Friesian (IF1, IF2, IF3 milk) cattle herds used in 12 Parmigiano Reggiano cheese-making trials ^1^.

Parameters	Unit	IB Milk		IF1 Milk		IF2 Milk		IF3 Milk		SE ^2^	*p* ^3^
		LSM		LSM		LSM		LSM			
Total Ca	mg/100 g	119.78	b	114.33	a	113.37	a	113.98	a	0.65	***
Soluble Ca	mg/100 g	36.72	b	35.62	a	34.74	a	34.69	a	0.55	*
Colloidal Ca	mg/100 g	83.05	b	78.71	a	78.63	a	79.29	a	0.65	***
Total P	mg/100 g	101.43	b	89.44	a	91.91	a	88.47	a	1.03	***
Soluble P	mg/100 g	44.02	b	37.67	a	37.79	a	36.2	a	1.13	***
Colloidal P	mg/100 g	55.54	b	50.02	a	52.29	ab	50.51	a	1.05	**
Inorganic colloidal P	mg/100 g	32.91		29.71		31.96		30.18		1.16	NS
Casein P	mg/100 g	22.63	b	20.31	a	20.33	a	20.33	a	0.56	**
Total Mg	mg/100 g	11.09	c	10.63	b	9.99	a	10.27	ab	0.11	***
Soluble Mg	mg/100 g	8.31	c	8.01	bc	7.57	a	7.75	ab	0.09	***
Colloidal Mg	mg/100 g	2.78	c	2.62	b	2.42	a	2.52	ab	0.03	***
Chloride	mg/100 g	93.73	a	96.65	ab	93.71	a	97.43	b	0.78	**

^1^ In each trial, four cheese-making trials were performed in parallel: three with milk collected from three Italian Friesian cattle herds (IF1, IF2 and IF3) (each cheese-making was made with the milk of a single herd) and one with milk coming from a single Italian Brown cattle herd (IB). ^2^ Standard error. ^3^ *p*-value: NS. *p* >0.05; * *p* ≤ 0.05; ** *p* ≤ 0.01; *** *p* ≤ 0.001; a, b, c different for *p* ≤ 0.05.

**Table 3:** Least square mean (LSM) values of pH, titratable acidy, rennet coagulation parameters, somatic cell count and total bacteria count of vat milk from Italian Brown (IB milk) or Italian Friesian (IF1, IF2, IF3 milk) cattle herds used in 12 Parmigiano Reggiano cheese-making trials ^1^.

Parameters	Unit	IB Milk		IF1 Milk		IF2 Milk		IF3 Milk		SE ^2^	*p* ^3^
		LSM		LSM		LSM		LSM			
pH	Value	6.72	a	6.74	ab	6.74	ab	6.76	b	0.01	*
Titratable acidity	°SH/50 mL	3.44	b	3.15	a	3.17	a	3.06	a	0.03	***
Clotting time	minutes	19.33		18.86		19.67		20.48		0.45	NS
Curd firming time	minutes	2.00	a	2.30	a	2.98	a	4.69	b	0.34	***
Curd firmness	mm	43.03	c	40.62	bc	34.49	b	26.19	a	1.66	***
Somatic cell count ^4^	10^3^ cells/mL	170	a	254	ab	160	a	282	b	24	**
Total bacterial count ^4^	10^3^ FCU/mL	51		23		60		30		11	NS

^1^ In each trial, four cheese-making trials were performed in parallel: three with milk collected from three Italian Friesian cattle herds (IF1, IF2 and IF3) (each cheese-making was made with the milk of a single herd) and one with milk coming from a single Italian Brown cattle herd (IB). ^2^ Standard error. ^3^ *p*-value: NS. *p* > 0.05; * *p* ≤ 0.05; ** *p* ≤ 0.01; *** *p* ≤ 0.001; a, b, c different for *p* ≤ 0.05. ^4^ Vat milk is composed (1:1) by the partially skimmed evening milk (by natural creaming) and the full-cream morning milk. As the natural creaming process lead to a marked reduction in somatic cells and bacterial count, these values were measured only on the full-cream morning milk.

**Table 4:** Least square mean (LSM) values of 24-h actual cheese yield, estimated cheese-making losses and curd fines of cheese made from Italian Brown (IB) or Italian Friesian (IF1, IF2, IF3). Results of 12 Parmigiano Reggiano cheese-making trials ^1^.

Parameters	Unit	IB Milk		IF1 Milk		IF2 Milk		IF3 Milk		SE ^2^	*p* ^3^
		LSM		LSM		LSM		LSM			
24-h Actual cheese yield ^4^	kg/100 kg	9.33	b	8.23	a	8.55	a	8.42	a	0.12	***
Estimated cheese-making losses ^5^											
Protein	%	26.71	a	27.19	ab	27.48	ab	27.93	b	0.24	**
Casein	%	1.25		1.72		1.33		1.48		0.25	NS
Fat	%	9.18	a	12.15	b	12.38	b	14.81	c	0.48	***
Curd fines	mg/kg	85.05	a	151.88	b	103.22	ab	117.00	ab	12.18	**

^1^ In each trial, four cheese-making trials were performed in parallel: three with milk collected from three Italian Friesian cattle herds (IF1, IF2 and IF3) (each cheese-making was made with the milk of a single herd) and one with milk coming from a single Italian Brown cattle herd (IB). ^2^ Standard error. ^3^ *p*-value: NS. *p* > 0.05; ** *p* ≤ 0.01; *** *p* ≤ 0.001; a, b, c different for *p* ≤ 0.05. ^4^ Measured 24 h after the extraction of the cheese mass from the vat. ^5^ Values expressed as follow: concentration in the residual cheese whey × 100/concentration in vat milk.

**Table 5:** Evaluation of the predicting ability of the cheese yield formula ^1^ developed here: comparison between actual (24-h ACY) and predicted cheese yield (24-h PCY) in calibration, validation and test phases.

Parameters	Unit	*n*	24-h ACY	24-h PCY	RMSEP ^2^	*p* ^3^
			Mean	Mean		
Calibration ^4^	kg/100 kg	36	8.38	8.38	1.53	NS
External validation ^5^	kg/100 kg	12	8.09	7.99	2.00	NS
Test ^6^	kg/100 kg	12	9.33	9.07	2.94	*

^1^ 24-h PCY = 1.254 × casein + 1.426 × fat + 1.059 (*R*^2^ 0.88; *p* ≤ 0.001) where casein and fat correspond to their contents (g/100 g) in vat milk. ^2^ Root mean standard error percentage. ^3^ *p*-value: NS. *p* > 0.05; * *p* ≤ 0.05. ^4^ Calibration of the formula was performed with data collected in Parmigiano Reggiano cheese-making trials carried out with IF1 (n. 12), IF2 (n. 12) and IF3 (n. 12). The cheese-making trials were carried out in parallel in the same cheese factory. ^5^ External validation of the formula was performed with data collected in 12 Parmigiano Reggiano cheese-making trials carried out in six different cheese factories. ^6^ Test of the formula was carried with data collected in 12 Parmigiano Reggiano cheese-making trials carried out with IB milk. These cheese-making trials were manufactured in parallel with those of IF1, IF2, and IF3 milk.
